# Signet Ring Cell Carcinoma of the Colon With Spermatic Cord Metastasis

**DOI:** 10.7759/cureus.83796

**Published:** 2025-05-09

**Authors:** Rin Morishita, Rina Fujiwara-Tani, Ruiko Ogata, Naokuni Tatsumoto, Hiroki Kuniyasu

**Affiliations:** 1 Molecular Pathology, Nara Medical University, Kashihara, JPN; 2 Surgery, Miyoshi Central Hospital, Miyoshi, JPN

**Keywords:** colonic signet ring cell carcinoma, metastasis, peritoneal dissemination, seminal cord metastasis, signet ring cell carcinoma

## Abstract

We report a case of metastatic signet ring cell carcinoma (SRC) of the colon involving the right spermatic cord and testis following a right hemicolectomy. A 53-year-old male presented to our hospital in August 2020 with abdominal distension. Colonoscopy revealed a stenotic tumor in the transverse colon, and curative right hemicolectomy was performed. Histopathological examination confirmed colorectal cancer (CRC), classified as Stage IIIc. Postoperatively, capecitabine was administered for six months. In October 2021, the patient revisited our hospital with right scrotal swelling and pain. Although tumor markers were negative, CT revealed a tumor in the spermatic cord. Subsequently, orchiectomy and spermatic cord excision were performed. Histopathological findings confirmed SRC, consistent with the primary colon cancer diagnosed at age 53, and the lesion was considered a metastasis. This case represents an extremely rare occurrence of CRC of the colon, further complicated by metastasis to the spermatic cord, an uncommon metastatic site for CRC.

## Introduction

Colorectal cancer (CRC) was the third most commonly diagnosed cancer worldwide in 2020, with over 1.9 million cases and approximately 930,000 deaths [1,2]. By 2040, due to population aging, the incidence is estimated to reach 3.2 million cases, with 1.6 million deaths, making it a significant global health concern [3].

Nearly all CRC cases are adenocarcinomas, with the majority being conventional adenocarcinomas. In contrast, signet ring cell carcinoma (SRC) accounts for only about 1% of CRC cases, making it extremely rare [4,5]. SRC is defined by the presence of tumor cells with signet ring morphology comprising at least 50% of the tumor, and it is recognized as a biologically aggressive subtype. Signet ring morphology describes cancer cells with a crescent-shaped, peripherally displaced nucleus due to intracellular mucin accumulation, giving the appearance of a signet ring. Compared to adenocarcinomas, SRC is characterized by a higher tumor grade and a more advanced stage at diagnosis [6-9]. Additionally, colorectal SRC commonly exhibits a high incidence of regional lymph node metastasis, lymphovascular invasion, and perineural infiltration [7,8]. Perineural invasion refers to the infiltration of cancer cells into the space surrounding nerves, serving as a route for tumor spread and often indicating aggressive behavior and poor prognosis. It also demonstrates a higher frequency of local and distant metastases, predominantly peritoneal dissemination, with a lower incidence of liver metastases [5]. Notably, peritoneal dissemination occurs in more than 50% of cases [7-9]. Peritoneal dissemination is the implantation and growth of tumor cells on the peritoneal surfaces.

On the other hand, malignant tumors account for 22% of spermatic cord tumors, and half of these are metastatic [10]. Spermatic cord metastasis is extremely rare, with metastases to the testis or peritesticular region from gastrointestinal cancers occurring in only approximately 0.3% of cases [11,12]. Among these, CRCs and gastric cancers most frequently metastasize to the spermatic cord [13]. Furthermore, spermatic cord metastasis is often associated with multiorgan metastases, with peritoneal dissemination observed in 65% of cases [14]. Thus, metastasis to the spermatic cord is rare, and the routes of metastasis are varied, including hematogenous, lymphatic, and peritoneal dissemination, making early detection difficult.

This case represents a rare occurrence of SRC of the colon metastasizing to the spermatic cord, an uncommon metastatic site for CRC.

## Case presentation

A 53-year-old male presented to our hospital in August 2020 due to progressive abdominal distension that had begun in early 2020. On physical examination, the abdomen was distended with excessive gas accumulation. Blood tests performed on September 12, 2020, revealed elevated white blood cell count and C-reactive protein levels (Table 1).

**Table 1:** Blood analysis. WBC: white blood cell; RBC: red blood cell; AST: aspartate aminotransferase; ALT: alanine aminotransferase; LDH: lactate dehydrogenase; BUN: blood urea nitrogen; CRP: C-reactive protein; CEA: carcinembryonic antigen; CA19-9: carbohydrate antigen 19-9

Parameter	Patient values		Reference range
Date	Day 8	Day 419	
WBC (/μL)	19,800	5,500	4,000–8,000
RBC (10^4^/μL)	450	522	380–480
Platelet (10^4^/μL)	36	23.4	15–35
Total protein (g/dL)	7.0	7.3	6.5–8.0
Albumin (g/dL)	4.1	4.3	4.1–5.1
AST (U/L)	15	24	13–37
ALT (U/L)	11	32	8–45
LDH (U/L)	197	233	118–335
Total bilirubin (mg/dL)	0.9	0.7	0.4–1.5
BUN (mg/dL)	21.0	12.0	8–20
Creatinine (mg/dL)	0.69	0.74	0.46–0.79
CRP (mg/dL)	2.2	0.1	0–0.3
CEA (mg/dL)	3.0	2.5	5–10
CA19-9 (U/L)	19.0	19.0	0–37

**Figure 1:**
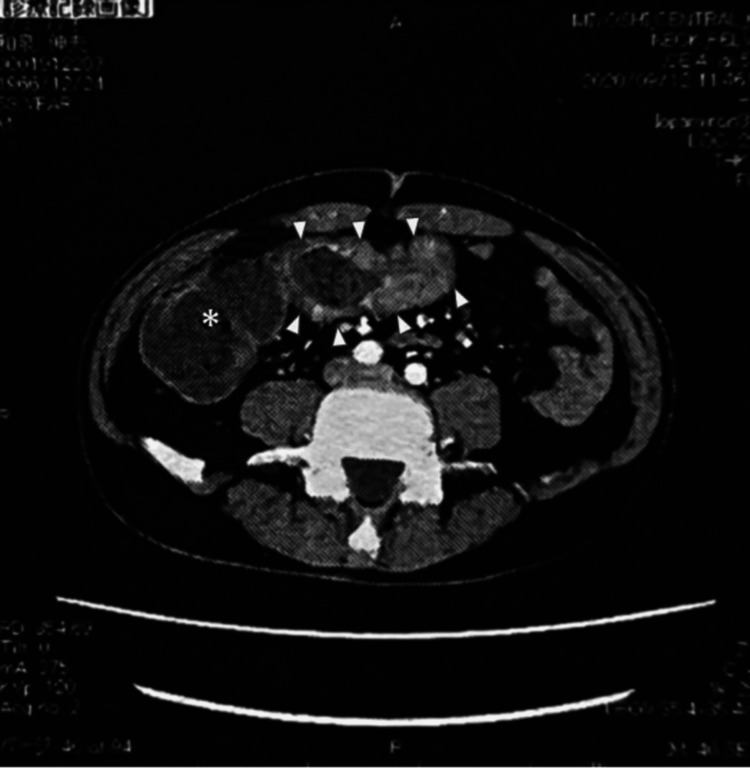
Abdominal CT scan. A circumferentially stenosing tumor 7 cm in length is observed in the transverse colon (arrows). The oral colonic lumen is dilated (asterisk)

An abdominal CT scan detected a 7 cm-long stenotic lesion in the transverse colon (Figure 1). Consequently, on day 11, an open right hemicolectomy with lymphadenectomy up to the D3 level was performed.

**Figure 2:**
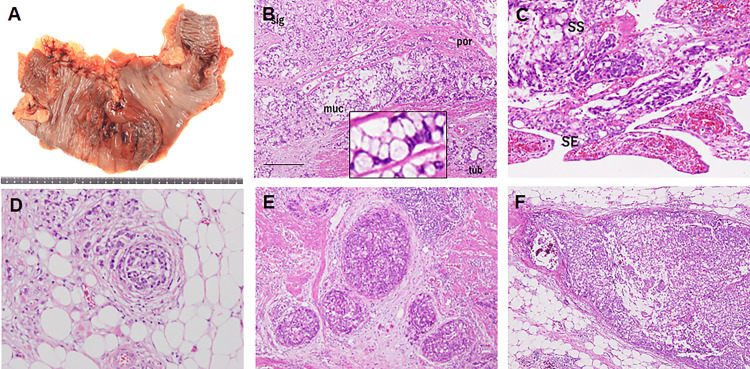
Pathological examination of colorectal cancer. (A) Macroscopic findings: a tumor measuring approximately 12 × 6 cm is observed in the hepatic flexure of the transverse colon. The surrounding mucosa exhibits diffuse edematous thickening. (B) Histopathological findings (hematoxylin and eosin staining, scale bar: 100 μm): proliferation of signet ring cell carcinoma is observed. Mucinous adenocarcinoma, moderately differentiated adenocarcinoma, and poorly differentiated adenocarcinoma are also present as minor populations. Inset, signet ring cells at high maginification. (C) Tumor invasion depth: cancer infiltrates from the mucosa to the subserosal layer and partially extends to the serosal surface. (D) Venous invasion. (E) Lymphatic invasion. (F) Metastasis to regional lymph nodes. sig: signet ring cell carcinoma; muc: mucinous adenocarcinoma; tub: moderately differentiated adenocarcinoma; por: poorly differentiated adenocarcinoma; SS: subserosal layer; SE: serosal exposure

Macroscopic examination of the resected specimen revealed a 12 × 6 cm tumor in the hepatic flexure. Unlike typical CRCs, this tumor exhibited minimal ulceration, with diffuse edematous thickening of the mucosa (Figure 2A). Histopathological analysis confirmed SRC, characterized by malignant cells with intracellular mucin accumulation and nuclear displacement. Additionally, poorly differentiated adenocarcinoma with invasive cord-like structures and a minor component of tubular adenocarcinoma were observed (Figure 2B). Tumor invasion extended widely to the serosal surface (Figure 2C). Furthermore, venous invasion (Figure 2D), lymphatic invasion (Figure 2E), perineural infiltration, and lymph node metastases (Figure 2F) were detected.

**Figure 3:**
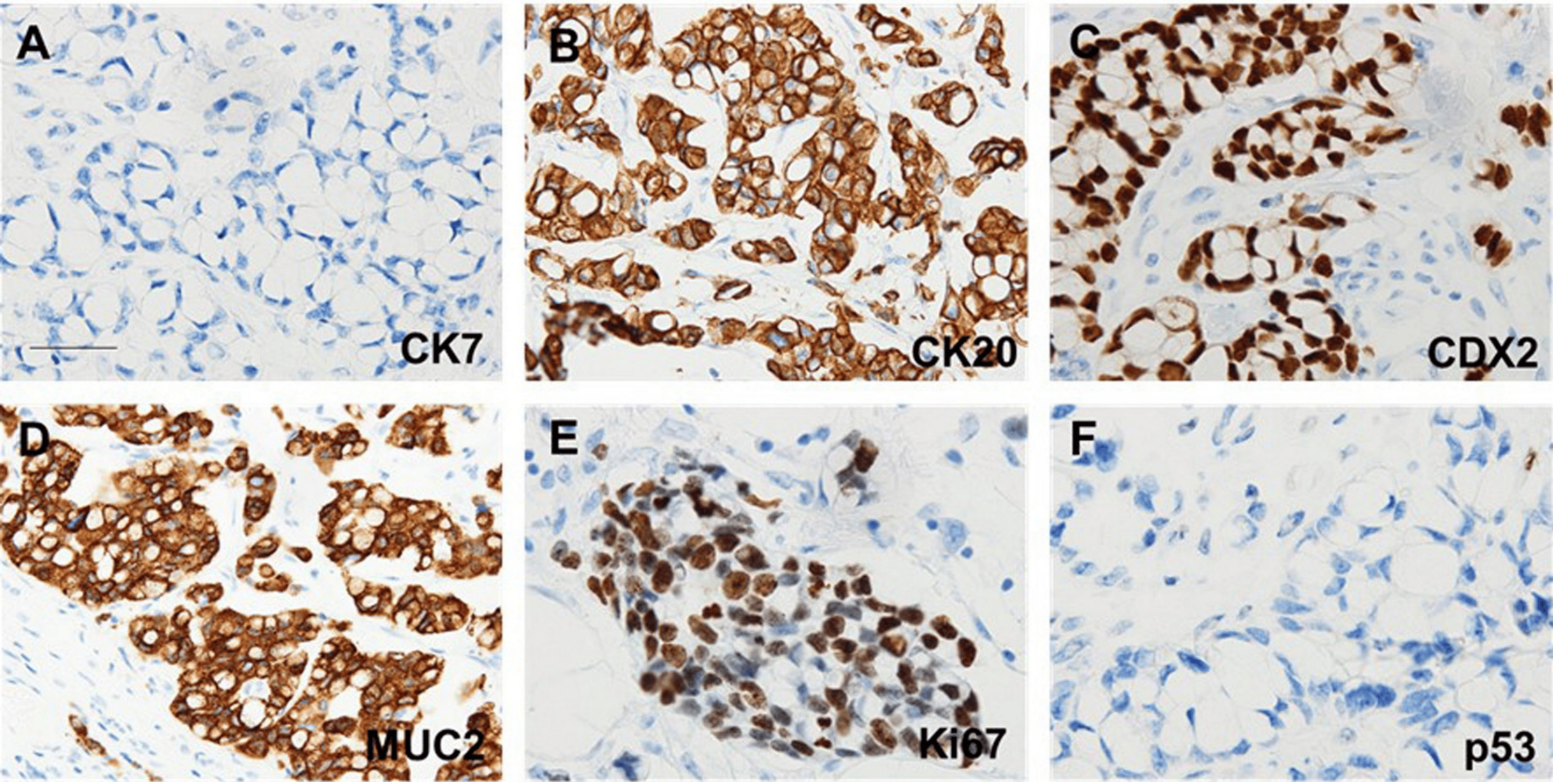
Immunohistochemical analysis of colorectal cancer. (A) CK7. (B) CK20. (C) CDX2. (D) MUC2. (E) Ki-67. (F) p53 (scale bar: 100 μm). Strong positive staining is observed for CK7, CDX2, and MUC2. CK: cytokeratin; CDX2: caudal-related homeodomain protein 2; MUC: mucin

Immunohistochemical analysis revealed that the tumor cells were positive for CK20, CDX2, and MUC2, with a Ki-67 proliferation index of 70%. CK7 and p53 were negative (Figure 3).

The final pathological diagnosis was adenocarcinoma (transverse colon, resected, 12 × 6 cm, sig>muc>por2, pT4a, Ly1c, V1c, Pn1a, budding grade 3, pPM0, pDM0, Stage IIIc) and metastatic carcinoma (lymph nodes, resected, positive node, 8/27, pN2b).

**Figure 4:**
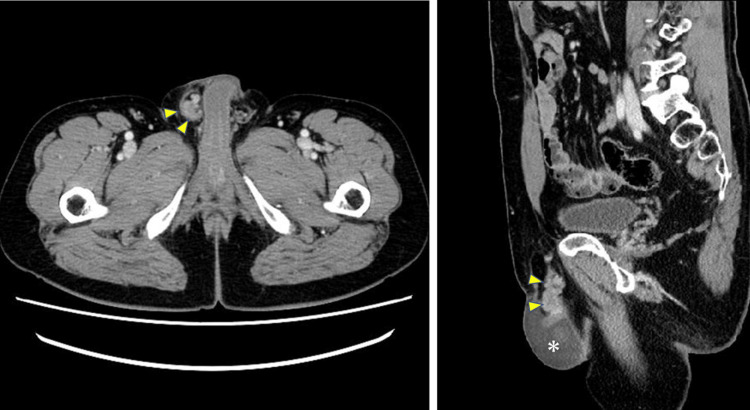
Abdominal CT scan. Left image: a tumor shadow is observed within the right swollen spermatic cord (arrows). Right image: a tumor shadow is observed within the right spermatic cord extending from the right inguinal canal to the epididymal region (arrows) with dilated processus vaginalis peritonei. A hydrocele is observed in the right scrotum (asterisk).

Postoperatively, the patient underwent adjuvant chemotherapy with capecitabine (3.6 g/day) for six months, starting on day 40. Subsequently, three cycles of XELOX (capecitabine (Xeloda) and oxaliplatin) therapy and nine cycles of mFOLFOX6 (modified folinic acid (Leucovorin), fluorouracil, and oxaliplatin) were administered. However, on day 416, he presented with right scrotal swelling and pain. Blood tests performed on day 419 showed mild elevations in alanine aminotransferase and lactate dehydrogenase levels, while tumor markers were negative (Table 1). An abdominal CT scan revealed a tumor within the right spermatic cord, along with scrotal hydrocele (Figure 4).

**Figure 5:**
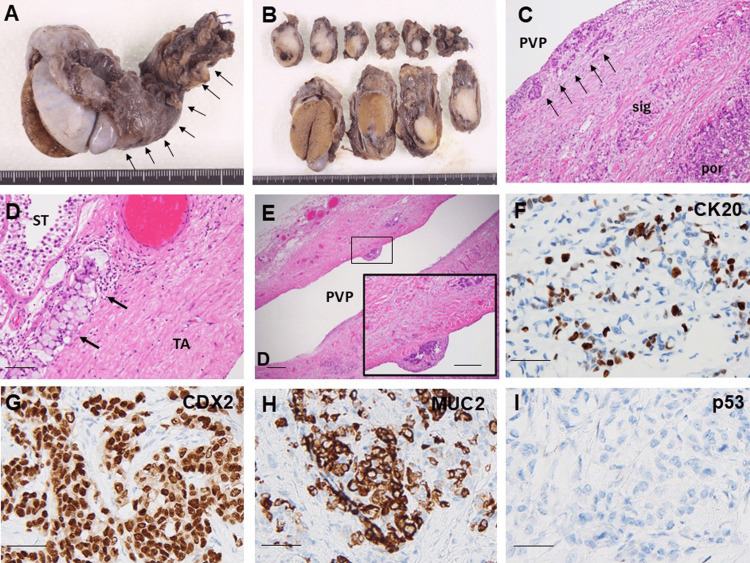
Pathological examination of the spermatic cord tumor. (A) Macroscopic findings: enlargement of the spermatic cord is noted (arrow). (B) Cross-section: a solid, white tumor is observed within the spermatic cord. (C) Histopathological findings: signet ring cell carcinoma and a portion of poorly differentiated adenocarcinoma are present in the spermatic cord. Cancer nests are exposed to the PVP (arrow). (D) Invasion of signet ring cell carcinoma into lymphatic vessels beneath the tunica albuginea of the testis (arrow). (E) Disseminated tumor foci observed along the inner surface of the PVP (inset: magnified view). (C-E: hematoxylin and eosin staining, scale bar: 100 μm). (F-I) Immunohistochemical analysis (scale bar: 100 μm). (F) CK20 is positive, but with lower expression compared to the primary tumor. (G, H) CDX2 and MUC2 are positive, similar to the primary tumor. (I) p53 remains negative, as observed in the primary tumor. PVP: processus vaginalis peritonei; sig: signet ring cell carcinoma; por: poorly differentiated adenocarcinoma; ST: seminiferous tubule; TA: tunica albuginea; CK: cytokeratin; CDX2: caudal-related homeodomain protein 2; MUC: mucin

Consequently, spermatic cord and testicular resection were performed on day 423. Macroscopic examination of the resected specimen revealed a 4 × 2.2 × 1.8 cm mass within the right spermatic cord. The cut surface showed a solid, white tumor occupying approximately 70% of the spermatic cord. No obvious tumor was observed within the testis (Figures 5A, 5B). Histopathological examination confirmed proliferation of SRC and poorly differentiated adenocarcinoma within the spermatic cord tumor (Figure 5C). The frequency of SRC was approximately 90% of cancer cells. Cancer invasion into lymphatic vessels within the testis was observed (Figure 5D), as well as dissemination along the processus vaginalis peritonei (Figure 5E). Immunohistochemical staining demonstrated that the spermatic cord tumor was positive for CK20, CDX2, and MUC2, and negative for CK7 and p53 (Figures 5F-5I).

These findings strongly suggested metastasis from the primary colon cancer. Notably, CK20 positivity was reduced, indicating decreased differentiation associated with metastasis. The final pathological diagnosis was metastatic adenocarcinoma (right spermatic cord, resected: 4 × 2.2 × 1.8 cm, Ly(+), V(+), margin: negative) and metastatic adenocarcinoma (right testis, resected).

**Figure 6:**
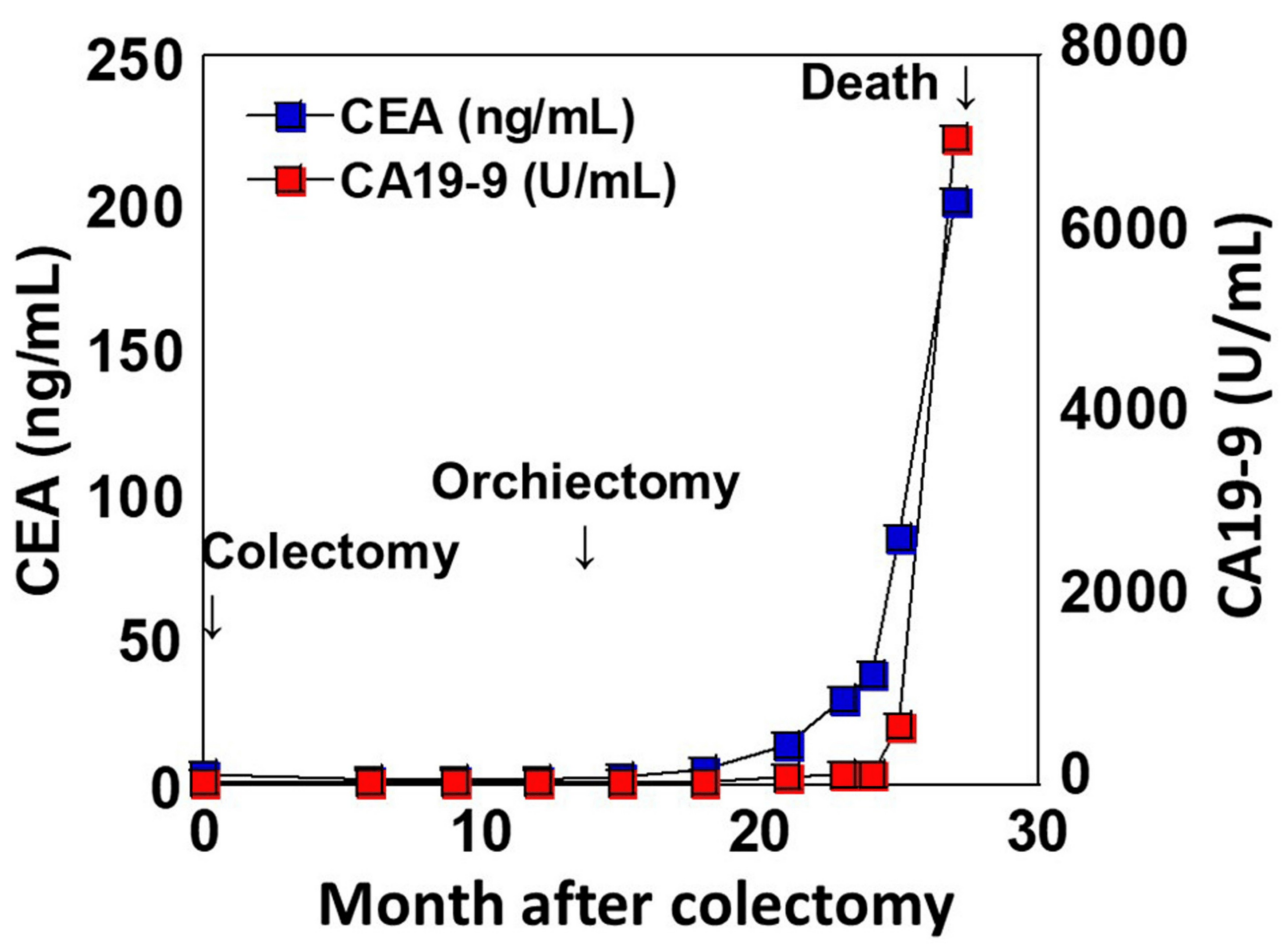
Changes in tumor markers. CEA and CA19-9 levels remained within the normal range before and after colectomy and spermatic cord/testis resection. However, following spermatic cord tumor resection, both markers showed a gradual increase. CEA: carcinembryonic antigen; CA19-9: carbohydrate antigen 19-9

Following spermatic cord tumor resection, serum levels of carcinoembryonic antigen and carbohydrate antigen 19-9 progressively increased, ultimately reaching 200.6 ng/mL and 7,114 U/mL, respectively. Treatment was not administered at the patient’s request, and he succumbed on day 783 to the disease, 27 months after the initial diagnosis and 12 months after spermatic cord and testicular resection (Figure 6).

## Discussion

In this case, SRC developed in the transverse colon and subsequently metastasized to the spermatic cord after surgical resection. While the majority of CRCs are adenocarcinomas, SRC accounts for fewer than 1% of CRC cases, making it extremely rare [4,5]. SRC is more commonly observed in gastric cancer; however, no gastric malignancy was detected in this case.

Colorectal SRC has distinct clinicopathological and molecular genetic characteristics compared to conventional adenocarcinomas [4]. It is more frequently diagnosed at a younger age and tends to occur predominantly in the right colon [4,6,7,9]. Unlike typical adenocarcinomas, which present as an intraluminal mass, SRC of the colon often exhibits a diffuse infiltrative growth pattern that mimics inflammatory diseases, leading to luminal stenosis. This characteristic increases the likelihood of false-negative results in endoscopic biopsies, and patients are frequently diagnosed at an advanced stage [4]. Additionally, colorectal SRC is known for its high malignancy potential, with a higher frequency of lymphatic, perineural, and vascular invasion. It also has a high incidence of peritoneal dissemination and exhibits metastases to unusual sites, such as bone, brain, bone marrow, ovaries, skin, and heart, which are rarely seen in conventional adenocarcinomas [4,7]. Due to its aggressive nature and poor prognosis, early detection is crucial. However, conventional imaging techniques such as CT and MRI may fail to detect SRC at an early stage. Therefore, the use of positron emission tomography-computed tomography and molecular markers, including circulating tumor DNA, at an early stage may improve early detection [4].

From a molecular pathology perspective, SRC exhibits a lower frequency of genetic mutations compared to adenocarcinomas [15]. While *RAS* mutations are common in conventional adenocarcinomas, SRC is characterized by a lower prevalence of *RAS* mutations and a higher frequency of sma- and mad-related protein (*SMAD*) mutations with loss of expression, *BRAF* mutations, microsatellite instability-high (MSI-H), and the CpG island methylator phenotype (CIMP) [4,15].

Chemotherapy is typically administered for SRC. In Stage III cases, 5-fluorouracil-based chemotherapy has been reported to be as effective as in conventional adenocarcinomas [9]. Chemotherapy is also effective against peritoneal dissemination [5,16]. In this case, capecitabine, XELOX, and mFOLFOX6 were administered following a right hemicolectomy. However, due to the patient’s discontinuation of medication, metastasis and recurrence could not be prevented. The prognosis of SRC is extremely poor, with a hazard ratio 1.5 times higher than that of adenocarcinomas [4,6,9].

This case presented with spermatic cord metastasis, which is extremely rare. Among primary tumors that metastasize to the spermatic cord and testis, gastric cancers and CRCs are the most common [13]. However, in CRC, the most frequent histological type of spermatic cord metastasis is moderately differentiated adenocarcinoma (37.5%), followed by poorly differentiated adenocarcinoma (25%) and mucinous adenocarcinoma (~10%), while SRC is not commonly observed [17]. Thus, this case of colonic SRC with spermatic cord metastasis is considered extremely rare.

Five potential metastatic pathways to the testis and spermatic cord from primary tumors have been reported, i.e., retrograde venous metastasis, arterial embolic metastasis, direct dissemination, retrograde vas deferens metastasis, and retrograde lymphatic spread [18,19]. Retrograde venous metastasis occurs when tumors, such as renal carcinoma, spread in a retrograde manner through the testicular vein, forming tumor thrombi in the pampiniform plexus. Arterial embolic metastasis occurs in gastrointestinal cancers, where tumor cells enter the liver via the portal circulation and subsequently disseminate. Direct invasion is thought to occur through peritoneal dissemination. Retrograde vas deferens metastasis is observed when primary tumors metastasize to the prostate or seminal vesicles and spread retrogradely through the prostate. Retrograde lymphatic spread occurs through the retroperitoneal lymph nodes.

In this case, the processus vaginalis peritonei remained continuous with the peritoneal cavity, and disseminated tumor foci were observed along its inner surface. Therefore, direct dissemination was considered the most likely metastatic pathway.

## Conclusions

This case highlights the aggressive nature of colorectal SRC, particularly its potential for uncommon metastatic spread, such as to the spermatic cord. Given the tendency of SRC to disseminate peritoneally and evade early detection by standard imaging or tumor markers, clinicians should maintain a high index of suspicion in patients with known SRC and new inguinal or scrotal symptoms. Surgical findings and histopathology remain essential for accurate diagnosis of such rare metastatic events. In cases where serosal invasion is evident, as seen in this patient, postoperative systemic chemotherapy is critical to disease control and recurrence prevention. Awareness of such atypical metastatic patterns may aid earlier diagnosis and inform management strategies in advanced colorectal SRC.
